# Pomegranate (*Punica granatum*) juice reduces oxidative injury and improves sperm concentration in a rat model of testicular torsion-detorsion

**DOI:** 10.3892/etm.2014.1782

**Published:** 2014-06-12

**Authors:** DOGAN ATILGAN, BEKIR PARLAKTAS, NIHAT ULUOCAK, YUSUF GENCTEN, FIKRET ERDEMIR, HUSEYIN OZYURT, UNAL ERKORKMAZ, HUSEYIN ASLAN

**Affiliations:** 1 Department of Urology, Faculty of Medicine, Gaziosmanpasa University, Tokat 60100, Turkey; 2 Department of Biochemistry, Faculty of Medicine, Gaziosmanpasa University, Tokat 60100, Turkey; 3 Department of Biostatistics and Medical Informatics, Faculty of Medicine, Sakarya University, Sakarya 54100, Turkey; 4 Department of Histology and Embryology, Faculty of Medicine, Gaziosmanpasa University, Tokat 60100, Turkey

**Keywords:** testis, torsion, ischemia reperfusion, oxidative stress, pomegranate juice

## Abstract

The present study aimed to evaluate the effect of pomegranate juice (PJ) on oxidative stress (OS) and sperm concentration in a rat model of testicular torsion-detorsion. A total of 21 Wistar albino rats were randomly divided into three groups, each consisting of seven rats, as follows: i) control group, which underwent sham surgery; ii) ischemia/reperfusion (I/R) group, designed to determine the effects of the testicular torsion-detorsion process on rats; and iii) PJ+I/R group, designed to evaluate the effect of PJ on the OS and sperm cell concentrations induced by the torsion-detorsion process. In the PJ+I/R group, the rats were given 0.4 ml/day PJ orally over a period of eight weeks prior to surgery. Ipsilateral orchiectomy was carried out and 5-cm^3^ blood samples were obtained from the inferior vena cava of all rats. Biochemical analyses were performed to calculate the superoxide dismutase (SOD) activity and malondialdehyde (MDA) levels in the testicular tissue and serum. The concentrations of spermatids, spermatocytes and spermatogonia in the seminiferous tubules were assessed using histopathological methods. Serum and tissue SOD and MDA levels were significantly higher in rats from the I/R group compared with the control group (P<0.001). PJ treatment significantly decreased the SOD and MDA levels in both the serum and testicular tissue of the rats (P<0.001). The spermatid, spermatocyte and spermatogonia concentrations were significantly reduced in the I/R group compared with the control group (P<0.001). PJ treatment significantly improved the concentrations of spermatids, spermatocytes and spermatogonia compared with those in the I/R group (P=0.008). The experimentally established testicular torsion-detorsion model led to OS in the rat testes. Daily consumption of PJ prior to surgery reduced OS parameters and improved sperm cell concentrations.

## Introduction

The pomegranate (*Punica granatum*) is a small, fruit-bearing tree that has been cultivated and naturalized since ancient times throughout the entire Mediterranean region. One of its extracts, pomegranate juice (PJ), is consumed around the world. A previous study revealed that PJ contains certain constituents that appear to have beneficial therapeutic properties, including antioxidative effects (1). Consequently, the protective effect of PJ consumption on various diseases is receiving considerable attention at present in all medical fields. The anticancer, antimicrobial, antiproliferative and cardioprotective effects of PJ and its metabolites have also been demonstrated in numerous previous studies (2–5). Pomegranate contains a number of polyphenols, including anthocyanins, minor flavonoids and punicalagin, which is the most important member of the ellagitannins family. The punicalagin is the largest polyphenol among the pomegranate ellagitannins and it is responsible from most of the antioxidant activity of the PJ (6). The potent antioxidant effects of polyphenols have been demonstrated in clinical and experimental studies (7,8). Thus, the pomegranate and its extracts have powerful antioxidant effects, which have also been revealed for other fruit juices, including grape, blackberry and branberry juice, and in green tea (9).

Testicular torsion is a rarely observed, serious urologic emergency, usually encountered in prepubertal males, which requires immediate surgical examination for proper treatment. It has been hypothesized that treatment for testicular torsion may lead to long-term infertility due to ischemia/reperfusion (I/R) injury which occurs following surgical correction of the torsed testis (10). Oxidative stress (OS) is a complex phenomenon that causes tissue damage and occurs due to an excessive production of reactive oxygen species (ROS) that are not adequately eliminated by the natural antioxidant defence mechanisms in the body. OS may cause significant damage to tissues by inducing differentiation in the cell membranes leading to irreversible cellular damage (11). In this context, OS increases the levels of malondialdehyde (MDA) and protein carbonyls (PCs) in the tissues, which are the end products of lipid peroxidation and protein oxidation, respectively.

Under physiological conditions, the enzymes belonging to the natural antioxidant defence system, including superoxide dismutase (SOD), catalase and glutathione peroxidase (GSH-Px), eliminate the ROS, which are released as a result of normal cellular metabolism (12). Excess levels of ROS particularly target unsaturated fatty acids, which play an important role in the constitution of cell membranes. Since the membranes of sperm contain a high abundance of unsaturated fatty acids (13), these cells are severely affected by peroxidation damage caused by ROS. Lipid peroxidation destroys the structure of the lipid matrix in the membranes of spermatozoa and is associated with the loss of motility and defects in membrane integrity (14).

In the present experimental study, a rat model of testicular torsion-induced OS was established. The biochemical and histopathological effects of daily PJ consumption on the OS parameters and sperm concentrations of the rats were subsequently evaluated.

## Materials and methods

### Study animals

Upon obtaining consent from the Gaziosmanpasa University Local Ethics Committee for Animal Experiments (Tokat, Turkey), 21 male Wistar albino rats (aged 5–6 months) were purchased Gaziosmanpasa University Experimental Medicine Research Center (Tokat, Turkey)for use in the present study. The experimental animals were housed at 18–22°C, under a 12 h light/dark cycle throughout the study period. Surgical procedures were performed under ketamine/xylazine anesthesia in sterile conditions. All rats were sacrificed following the experimental procedures.

### Group allocation

The rats were randomly divided into three groups, each group consisting of seven rats, as follows: i) control group, which underwent a sham surgical procedure to determine the basal values for biochemical and histopathological evaluations; ii) I/R group, designed to study the effects of the testicular torsion-detorsion process on the ipsilateral testicle and; iii) PJ+I/R group, designed to determine the effect of PJ on OS indicators and sperm parameters following unilateral testicular torsion-detorsion. In the PJ+I/R group, the rats were given 0.4 ml/day commercially-available PJ (Ersu® Fruit and Food Industries Inc., Konya, Turkey) orally over a period of eight weeks prior to surgery.

### Surgical procedure

In the control group rats, the testicle was exposed through a midline incision, a 4-0 silk suture was fixed through the tunica albuginea and the testicle was replaced into the scrotum with no additional intervention. In the rats in the I/R and PJ+I/R groups, the tunica vaginalis was opened and the right testis exposed to allow surgical intervention. It was rotated 720° in a clockwise direction and maintained in this torsed position by fixing the testicle to the scrotum with a 4-0 silk suture. After 1 h, the spermatic cord was detorsed and orchiectomy was performed on the right side. The resected testicular tissue specimens were separated into two parts for histopathological (sperm parameters) and biochemical (tissue MDA and SOD level) analyses. Furthermore, ~5-cm^3^ blood samples were obtained from the inferior vena cava of the rats to determine the blood levels of MDA and SOD.

### Biochemical analyses

Blood samples were drawn into heparin-free tubes for biochemical analysis. Following centrifugation (2,000 × g for 15 min at 4°C), the serum samples were frozen and stored at −70°C until required. Commercially-available chemical supplies (Sigma; St. Louis, MO, USA) were used for the determination of the following parameters in the serum samples.

### Serum antioxidant enzyme analysis

Total (Cu-Zn and Mn) SOD [enzyme commission (EC) number 1.15.1.1) activity was determined according to a previous method described by Sun *et al* (15). The principle of the method is based on the inhibition of nitroblue tetrazolium (NBT) reduction by the xanthine-xanthine oxidase system as a superoxide generator. Activity was assessed in the ethanol phase of the supernatant following the addition of 1.0 ml ethanol/chloroform mixture (5:3, v/v) to the same volume of sample and centrifugation (1,500 × g for 10 min). One unit of SOD was defined as the amount causing a 50% inhibition of the NBT reduction rate. The SOD activity was expressed in U/ml.

### Determination of the levels of MDA

The level of tissue thiobarbituric acid reactive substance (TBARS) was determined by a method based on a reaction with thiobarbituric acid (TBA) at 90–100°C that was previously described by Esterbauer and Cheeseman (16). In the TBA test reaction, MDA or MDA-like substances and TBA react to produce a pink pigment with an absorption maximum at 532 nm. The reaction was performed at pH 2–3 and 90°C for 15 min. The sample was mixed with a double volume of cold 10% (w/v) trichloroacetic acid to precipitate the protein. The precipitate was pelleted by centrifugation (1,500 × g for 10 min) and an aliquot of the supernatant was reacted with an equal volume of 0.67% (w/v) TBA in a boiling water bath for 10 min. Following cooling, the absorbance at 532 nm was measured (GBC Cintra 10e UV/VIS Spectrophotometer, Victoria, Australia). Results are expressed in ng/ml, according to the graphic standard prepared from measurements with a standard solution (1,1,3,3-tetramethoxypropane).

### Histological examination

The rat testes were fixed in Bouin’s solution (Polysciences Europe GmbH, Eppelheim, BadenWürttemberg, Germany) and passed through an increasing alcohol series. The testicular tissue was processed for paraffin embedding following treatment with xylene and paraffin. Subsequently, 5- and 20-μm-thick sections were obtained with a rotary microtome (LeicaRM 2135, Leica Instruments, Nussloch, Germany). The sections were randomly sampled and stained with Periodic acid-Schiff (PAS). Finally, the spermatogenic cells in the seminiferous tubules, including the spermatids, spermatocytes and spermatogonia, were counted with an optical fractionator (Stereology workstation: Trinokulermicroskop Leica DM 2500, Leica Instruments; motorizedstage: Ludlelectronics, New York, NY, USA; digitalmicrocator: Heidenhain, Traunreut, Germany, StereoInvestigator: MBF Biosciences, Williston, VT, USA). The spermatogenic cell concentrations are expressed in number of cells/mm3).

### Statistical analysis

Kruskal-Wallis one-way analysis of variance was used to compare the biochemical and sperm parameters among groups. For multiple comparisons, the Bonferroni adjusted Mann-Whitney U test was performed. Data are expressed as the median and interquartile range (IQR). P<0.05 was considered to indicate a statistically significant difference. Analyses were carried out using a commercially-available software (IBM SPSS Statistics 19; IBM, Armonk, NY, USA).

## Results

### SOD activity and MDA levels in the serum and testicular tissue

The levels of MDA and SOD in the serum and tissue samples of the three groups of rats are presented in Table I. No statistically significant differences were identified between the groups in terms of testicular tissue weights. In the I/R group, the SOD activity and MDA levels in the serum and testicular tissue were significantly higher compared with those in the control group (P<0.001). The consumption of PJ significantly reduced the SOD activity and MDA levels in the serum and tissue compared with those in the I/R group (P<0.001).

### Spermatogenic cell concentrations

The comparison of spermatogenic cell concentrations in the three groups of rats is presented in Table II. The concentrations of spermatids, spermatocytes and spermatogonia were significantly reduced in the I/R group compared with those in the control group (P<0.001). However, the consumption of PJ over a period of eight weeks significantly improved the spermatid, spermatocyte and spermatogonia concentrations compared with those in the I/R group (P=0.008).

### Histopathological analysis

**Figure 1:**
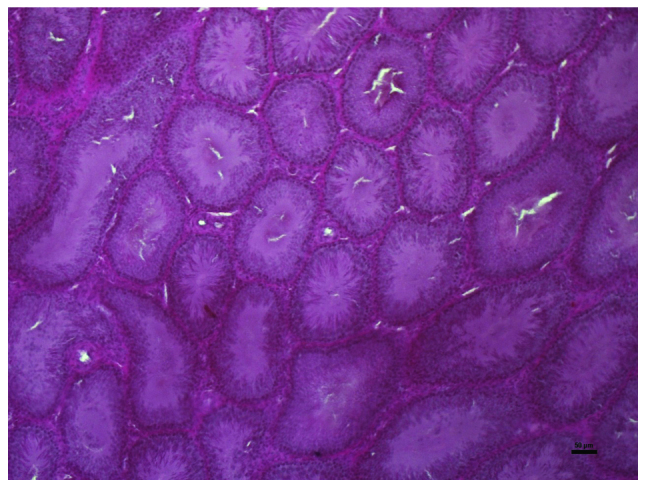
In the control group, the germ cells in seminiferous tubules were observed in regular form. Magnification, ×10.

**Figure 2:**
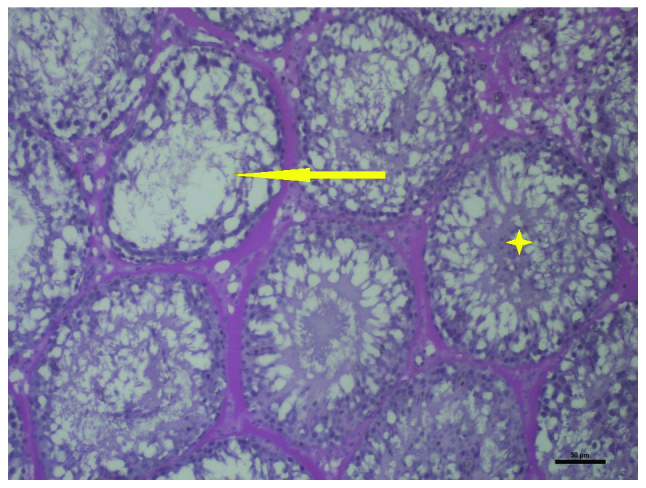
In the ischemia/reperfusion group, intensive vacuolization was observed in the seminiferous tubules (yellow arrow). Small numbers of spermatids, spermatocytes and spermatogonia were identified in the adluminal compartment, with irregularities in their germ cell sequences (yellow star). Magnification, ×20.

**Figure 3:**
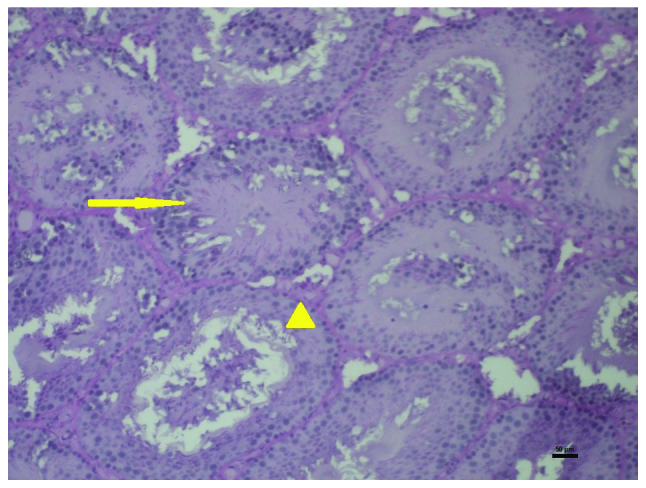
In the pomegranate juice + ischemia/reperfusion group, vacuolization and irregularities were observed in the seminiferous tubules (yellow arrowhead). However, similarly to the control group, a regular formation of germ cells was also detected in numerous seminiferous tubules (yellow arrow). Magnification, ×20.

In the histopathological analysis of the testicular tissues, intensive vacuolization with irregularities in the germ cell sequences were observed in the I/R group. A reduced incidence of vacuolizations and regular germ cell formation, comparable to that observed in the control group, were present in the PJ+I/R group. Representative microscopic images of the testicular tissues in the three groups are presented in Figs. 1–3.

## Discussion

Since it contains a high amount of polyphenols, PJ possesses strong antioxidant properties. The antioxidant effects of polyphenols, particularly ellagitannins, have been previously demonstrated in numerous studies (17–19). Ellagitannins and other polyphenols are hydrolyzed into ellagic acid (EA), which is responsible for their antioxidant activities *in vivo* (20). An *in vitro* study has revealed the ability of EA to easily pass through the mitochondrial membrane (21). In the present study, testicular torsion-detorsion caused elevations in the levels of SOD and MDA, which are the indirect indicators of oxidative injury. However, pretreatment with PJ over eight weeks decreased the levels of SOD and MDA in the serum and testicular tissue.

Under normal conditions, sperm cells produce high amounts of ROS as a result of their physiological metabolism (22). As testicular tissue contains a high amount of unsaturated fatty acids, it is very sensitive to the detrimental effects of ROS. Lipid peroxidation is able to damage the structure of the lipid matrix in sperm membranes, decrease intracellular levels of ATP leading to reduced sperm viability, cause axonemal damage and increase mid-piece morphological defects (23. However, testicular tissue contains numerous antioxidant enzymes and ROS scavengers that protect its spermatogenic function from OS and in particular from peroxidative damage, which is the most important cause of spermatogenic dysfunction (24,25). This antioxidant defence system scavenges considerable numbers of ROS in order to protect sperm cells from their harmful effects and preserves only a small number of ROS to maintain normal cell function. Without this defence system, the ROS cause thinning of the germ layer, decreased spermatogenic cell density and sperm motility, and an increased production of abnormal sperm cells (26).

The current study demonstrated that testicular torsion-detorsion induced OS in testicular tissue and caused a reduction in the concentrations of all spermatogenic cell types, including spermatogonia, spermatocytes and spermatids. The reduction in spermatogenic cell concentration, which occurred without any reduction in testicular tissue weight, may indicate that OS was the most important reason for this pathology. However, the daily consumption of PJ over a period of eight weeks prior to the torsion-detorsion surgery resulted in a reduction in the OS parameters and a significant increase in the spermatogenic cell concentration.

Similarly, in a previous experimental study, Türk *et al* revealed that the daily consumption of PJ over a period of seven weeks caused a reduction in OS parameters and a marked increase in the levels of spermatogenic cells and the thickness of the germ layer (27). Furthermore, Mansour *et al* demonstrated that the daily consumption of PJ at various doses for a period of six weeks resulted in a significiant increase in serum glutathione peroxidase (GSH-Px) and catalase activities in rats. The study also noted that PJ consumption significantly increased epididymal sperm concentration and sperm motility parameters and decreased abnormal sperm production (28).

In the current experimental study, it was indicated that testicular torsion-detorsion caused OS, which may have led to reduced sperm concentrations in the rat testes. However, the daily consumption of PJ prior to the torsion-detorsion surgery resulted in a reduction in the parameters of OS and produced an increment in spermatogenic cell concentrations.

## Figures and Tables

**Table I:** Levels of superoxide dismutase (SOD) and malondialdehyde (MDA) in the serum and testicular tissue of rats from the different groups.

Variable	Control	I/R	PJ+I/R	P-value
tSOD (U/ml)	13.18 (7.86–17.54)	29.37 (26.45–38.26)^a^	12.04 (8.61–13.66)	<0.001
tMDA (ng/ml)	86.09 (66–139.71)	270.37 (237.86–316.39)^a^	81.84 (70.08–103.53)	<0.001
sSOD (U/ml)	26.76 (23.67–29.52)	73.45 (61.66–81.47)^a^	22.1 (16.9–25.2)	<0.001
sMDA (ng/ml)	1660.6 (1589.3–1747.0)	2605.0 (2518.0–2894.8)^a^	1592.3 (1467.8–1706.6)	<0.001
Tissue weights (g)	0.23±0.03	0.23±0.02	0.23±0.03	0.860

Data are expressed as median (interquartile range).

a Significantly different from the control and PJ+I/R groups;

I/R, ischemia/reperfusion; PJ, pomegranate juice; t, tissue; s, serum; SOD, superoxide dismutase; MDA, malondialdehyde.

**Table II:** Comparison of the concentrations of spermatogenic cells between groups.

Cell type	Control	I/R	PJ+I/R	P-value
Spermatids	32.22 (28.35–36.35)	21.04 (17.16–28.01)^a^	28.68 (24.86–37.24)	P<0.001
Spermatocytes	18.56 (15.53–21.27)	11.53 (8.89–14.57)^a^	16.22 (13.84–18.39)	P<0.001
Spermatogonia	8.48 (6.89–10.06)	6.09 (4.10–7.35)^a^	7.81 (6.41–9.28)	P=0.008

Data are expressed as median (interquartile range) ×10^6^.

a Significantly different from the control and PJ+I/R groups;

I/R, ischemia/reperfusion; PJ, pomegranate juice.
